# Benefits of Biosimilars in the Management of Patients with Inflammatory Bowel Disease: An International Survey

**DOI:** 10.3390/jcm13113069

**Published:** 2024-05-24

**Authors:** Ferdinando D’Amico, Laurent Peyrin-Biroulet, Silvio Danese

**Affiliations:** 1 Department of Gastroenterology and Endoscopy, IRCCS San Raffaele Hospital, 20132 Milan, Italy; damico_ferdinando@libero.it; 2 Department of Gastroenterology, Nancy University Hospital, F-54500 Vandœuvre-lès-Nancy, France; peyrinbiroulet@gmail.com; 3 Inserm, NGERE, University of Lorraine, F-54000 Nancy, France; 4 INFINY Institute, Nancy University Hospital, F-54500 Vandœuvre-lès-Nancy, France; 5 FHU-CURE, Nancy University Hospital, F-54500 Vandœuvre-lès-Nancy, France; 6 Groupe Hospitalier Privé Ambroise Paré-Hartmann, Paris IBD Center, F-92200 Neuilly sur Seine, France; 7 Division of Gastroenterology and Hepatology, McGill University Health Centre, Montreal, QC H4A 3J1, Canada; 8 Department of Gastroenterology and Endoscopy, Vita-Salute San Raffaele University, 20132 Milan, Italy

**Keywords:** ulcerative colitis, Crohn’s disease, inflammatory bowel disease, advanced therapy, biosimilar

## Abstract

**Background/Objectives**: The development of biosimilar drugs has revolutionized the management of patients with inflammatory bowel diseases (IBD), significantly reducing healthcare costs. However, the impact of biosimilar availability on patient care is unknown. We conducted a survey to investigate the benefits of using biosimilars in patients with IBD. **Methods**: Physicians involved in the IBD care were invited to participate in an anonymous online survey. The questionnaire consisted of 42 questions addressing availability, cost, recommendations, and positioning regarding the use of biosimilars. **Results**: A total of 233 physicians (88.4% gastroenterologists) from 63 countries worldwide participated in the survey. Most respondents had >10 years of practice (202/233, 85.9%). Biosimilars were available in almost all cases (221, 94.8%), and over two-thirds of respondents had more than one biosimilar of adalimumab or infliximab on hospital formulary. In most cases, adalimumab and infliximab biosimilars had a reduced cost of at least 30% compared to the originators. The savings resulting from the use of biosimilars allowed physicians to improve patient care (3/233, 1.3%) or to improve research (2/233, 0.8%) in only a few cases. Interestingly, for about 50% of respondents, the cost of biologics was a limitation for patient access to therapy. For the majority of participants, the availability of biosimilars did not influence treatment decisions in Crohn’s disease (70/165, 42.4%) and ulcerative colitis (83/165, 50.3%). **Conclusions**: The reduced cost of biosimilars compared to reference products is the main driver of choice in IBD. The impact of biosimilars of ustekinumab and vedolizumab in improving access to therapies and changing the treatment algorithm remains to be defined.

## 1. Introduction

Crohn’s disease (CD) and ulcerative colitis (UC) are inflammatory bowel diseases (IBD) that have a significant burden on both patients and healthcare [1,2]. They are progressive diseases, and if not adequately controlled, expose patients to a high risk of hospitalization, surgery, and colorectal cancer [3,4]. The development of biologic drugs has revolutionized the management of IBD patients by reducing the rate of negative outcomes [5]. Specifically, infliximab and adalimumab, two tumor necrosis factor alpha (TNFα) inhibitors, were the first monoclonal antibodies approved for the management of patients with moderate to severe CD and UC who were unresponsive to conventional therapies [6,7]. These drugs have proven their effectiveness and safety in different settings, including pregnancy, pediatric patients, the elderly, perianal disease, and extraintestinal manifestations, and are still considered one of the main therapeutic options for the management of IBD [8,9,10]. However, their considerable cost has limited their widespread use and still influences therapeutic choices. In this context, the development of biosimilars, drugs which are proven to be as effective and safe as the originators but with a cheaper cost, has represented a key incentive for the widespread use of biological therapies [11]. Several studies have confirmed the efficacy and safety of biosimilars, supporting their choice in clinical practice [11,12,13]. To date, only biosimilars of adalimumab and infliximab are available, but soon the patents of other drugs (e.g., ustekinumab and vedolizumab) will expire and new biosimilar drugs will be available, impacting clinicians’ therapeutic choices [14]. To date, little is known about the impact of biosimilars on patient care. For this reason, we conducted an international survey to investigate the benefits of using biosimilars and to better understand whether the availability of biosimilars improves access to advanced therapies.

## 2. Materials and Methods

The survey aimed to gather information from clinicians worldwide who are involved in IBD care. The survey was conducted from November to December 2023, utilizing an online platform for data collection. We followed the Consensus for Reporting of Survey Studies (CROSS) guidelines to ensure transparency and consistency in reporting [15]. Survey invitations were disseminated through the mailing lists of IBD-scope, a webinar platform for healthcare professionals interested in IBD [16]. Screening questions were incorporated at the beginning of the survey to ensure that respondents belonged to the target population. Additionally, email registration was employed to prevent duplicate responses. Responses were collected anonymously to encourage honest and uninhibited feedback. Permission for data collection was obtained from all participants at the outset of the survey. Before starting the electronic survey, each participant was asked to explicitly give consent to the use of the survey data. Both the survey and invitation emails were in English, enabling a wider reach. The survey questions were structured as multiple choice, likely enhancing ease of response and subsequent data analysis. The questionnaire consisted of 42 questions. Demographics, specialty, and level of experience of respondents were collected. The survey focused on the use of biosimilars, including the availability of biosimilars in the center, their cost, recommendations from the hospital or regulatory authorities regarding their use, the impact of the availability of biosimilars on therapeutic choices, and the positioning of drugs in the treatment algorithm. The number of respondents for each question was reported to account for missing data, ensuring transparency in reporting. This study was conducted in accordance with the principles of the Declaration of Helsinki. The survey was noninterventional and was not intended to provide clinical data for treatment decisions; ethical approval was therefore not required. Informed consent was also not required, as all data were completely anonymized [17]. 

## 3. Results

### 3.1. Demographics

A total of 233 physicians from 63 countries worldwide participated in the survey. The most represented countries were Italy (39/233, 16.7%), Brazil (13, 5.6%), Belgium (8, 3.4%), Egypt (7, 3.0%), France (7, 3.0%), and Spain (7, 3.0%) (supplementary data). Gastroenterologists were the most represented specialists (206, 88.4%), followed by internists (7, 3.0%), pediatric gastroenterologists (6, 2.6%), surgeons (6, 2.6%), and other healthcare professionals (8, 3.4%) (Table 1). Most respondents had >10 years of practice (202/233, 85.9%) and a lot of experience in the field of IBD (more than 10 years in 179 cases, 76.8%, or 5–9 years in 32 cases, 13.7%). The number of IBD patients seen per year ranged from <100 (49, 21.0%) to <500 (93, 39.9%) to <1000 (45, 19.3%). Only a fifth of physicians saw >1000 patients per year (32, 13.7%) or >2000 patients per year (14, 6.0%). Approximately three-quarters of respondents (171, 73.4%) saw <50 new/recently diagnosed IBD patients per year, while only a small percentage visited 51–100 patients per year (39, 16.7%) or >100 new patients per year (23, 9.9%).

### 3.2. Availability of Biosimilars

Almost all respondents (221, 94.8%) had biosimilars available in their clinical practice. In most cases (192/221, 86.9%), both infliximab and adalimumab biosimilars were available, while only a few practitioners had only adalimumab (16, 7.2%) or only infliximab (13, 5.9%). Among the 5.2% of physicians who did not have access to the biosimilars, the reasons for the unavailability include the following: the center does not purchase them (10/12, 83.4%), no cost difference compared to the originator (1, 8.3%), and a lack of confidence in the efficacy of the biosimilar (1, 8.3%). Interestingly, most clinicians had more than one adalimumab (161/208, 77.4%) or infliximab (138/205, 67.3%) biosimilar available. The main criteria for choosing one biosimilar over the other were availability in the pharmacy (30/208, 14.4%), cost (27, 13.0%), device characteristics (16, 7.7%), hospital indications (14, 6.7%), and insurance recommendations (9, 4.3%).

### 3.3. Cost of Biosimilars

**Figure 1:**
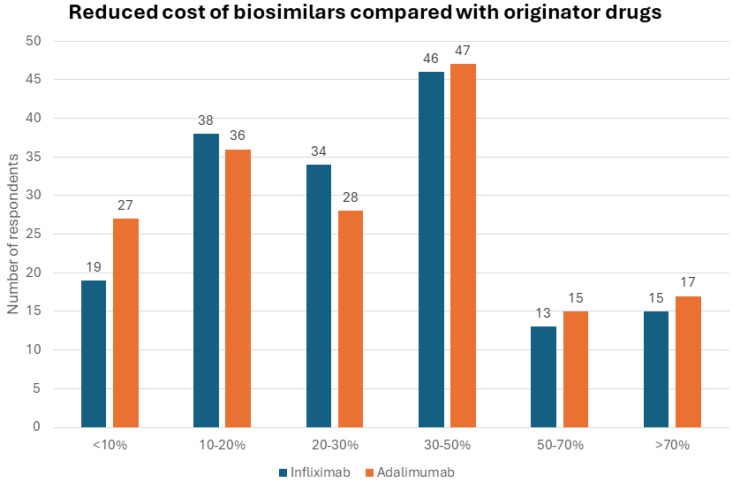
Reduced cost of biosimilars compared with originator drugs.

For half of the respondents (121, 51.9%), the cost of biologics was a limitation for patient access to therapy. Adalimumab biosimilars had a reduced cost compared to the originator for the majority of those who had adalimumab biosimilars available (170/208, 81.7%). Specifically, there was a cost reduction of at least 30% in approximately half of the cases: a 30–50% discount (47/170 cases, 27.6%), a 50–70% discount (15 cases, 8.8%), and a >70% discount (17 cases, 10%) (Figure 1). Similarly, infliximab biosimilars had a reduced cost compared to the originator for most participants (165/205, 80.5%). Again, the infliximab biosimilar cost at least 30% less than the originator in about half of the cases: a 30–50% discount (46/165, 27.9%), a 50–70% discount (13, 7.9%), and a >70% discount (15, 9.1%). 

**Figure 2:**
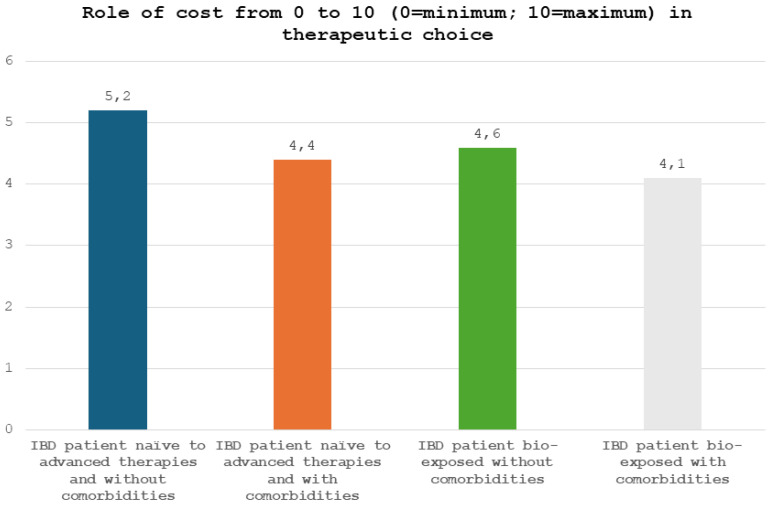
Role of cost, from 0 to 10 (0 = minimum; 10 = maximum), in therapeutic choice.

We asked physicians to quantify, from 0 to 10 (0 = minimum; 10 = maximum), the impact of drug costs on their choice of therapy. In an IBD patient naïve to advanced therapies and without comorbidities, cost had a marginal role in the physicians’ therapeutic decision between an adalimumab/infliximab biosimilar and an originator drug with another mechanism of action (e.g., ustekinumab or vedolizumab) (mean value, 5.2 ± 3.6) (Figure 2). Importantly, in an IBD patient naïve to advanced therapies and with comorbidities, the cost had an even more marginal role in the therapeutic decision between an adalimumab/infliximab biosimilar and an originator drug with another mechanism of action (e.g., ustekinumab or vedolizumab) (mean value, 4.4 ± 3.4). Similarly, in a patient with IBD already treated with advanced therapies, cost had a limited impact on the therapeutic decision between an adalimumab/infliximab biosimilar and an originator drug with another mechanism of action (e.g., ustekinumab or vedolizumab) regardless of comorbidities (mean value, 4.6 ± 3.3 without comorbidities; mean value, 4.1 ± 3.4 with comorbidities).

### 3.4. Recommendations about Biosimilars

About a third of the respondents (90/233, 38.6%) received recommendations from the hospital pharmacy regarding the prescription of biosimilars or originator drugs. Almost all clinicians (89/90, 98.9%) received a recommendation to use the biosimilar with the lowest cost, while only in one case the originator drug was suggested (1/90, 1.1%). A small number of respondents received recommendations regarding the prescription of biosimilars or originator drugs from their local regulatory authority (45/233, 19.3%). In all cases, the use of the biosimilar was recommended. In only a limited proportion of cases, departments received a refund that could be used for patient care (3/233, 1.3%) or to improve research (2/233, 0.8%) if the clinician used biosimilars instead of originator drugs.

### 3.5. Patient Knowledge and Benefits of Biosimilars 

There was high variability in the percentage of patients who were aware of biosimilars: <10% (47/233, 20.2%), 11–25% (37, 15.9%), 26–50% (43, 18.4%), 51–75% (39, 16.7%), >75% (38, 16.3%), or not known (29, 12.5%). The main source of patient awareness regarding biosimilars were physicians (87/233, 37.3%), followed by the Internet (30, 12.9%), a patient association (10, 4.3%), and IBD nurses (8, 3.4%). According to the respondents, the potential benefits of biosimilars for IBD patients were reduced healthcare costs (112/223, 50.2%), improved access to treatment (60, 25.7%), increased competition leading to improved innovation (20, 8.9%), and increased treatment options (12, 5.1%). Most participants explained the benefits of biosimilars to their patients systematically (73/233, 31.3%), often (57, 24.5%), or sometimes (59, 25.3%). On the other hand, patients’ understanding of the benefits of biosimilars, from 0 to 10 (0 = very little; 10 = fully), was mostly adequate: 0–2 (8/189, 4.2%), 3–6 (56, 29.6%), and 7–10 (125, 66.2%). For approximately half of the respondents, the availability of biosimilars did not allow more patients to be treated in an earlier stage of their disease (125/233, 53.6%).

### 3.6. Positioning of Biosimilars in the Therapeutic Algorithm

The switch to the biosimilar was mostly performed at any time (on medical indication) (66/221, 29.9%) or was not a medical decision (51, 23.1%). It was also performed when the patient achieved remission (29, 12.5%) or when the originator drug was not available (7, 3.2%). For the majority of participants, the availability of biosimilars did not influence treatment decisions in CD (70/165, 42.4%) or in UC (83/165, 50.3%). In a third of cases, biosimilars influenced therapeutic decisions both in CD and UC by allowing early treatment (39, 23.6% and 38, 23.0%), reducing costs (13, 7.9% and 13, 7.9%), or modifying the positioning of drugs in the therapeutic algorithm (5, 3.0% and 5, 3.0%). In most cases, disease characteristics did not influence the decision to use a biosimilar over the originator drug (134/221, 60.6%). However, in a small proportion of cases, previous treatments (20, 9.0), disease severity (18, 8.1%), the presence of complications (15, 6.8%), disease location (7, 3.2%), and disease duration (7, 3.2%) could have had a role in the therapeutic choice.

### 3.7. Impact of New Upcoming Biosimilars on Therapeutic Decisions

For a significant portion of physicians, the availability of ustekinumab and vedolizumab biosimilars in the near future will not increase access to therapies (45/233, 19.3%), or their impact is not known (117, 50.2%). Instead, for the remainder, the availability of ustekinumab and vedolizumab biosimilars could increase access to therapies, allowing for early treatment (32, 13.7%), reduced costs (19, 8.1%), and a new positioning of these drugs in the therapeutic algorithm (13, 5.6%). Likewise, for a relevant proportion of subjects, it was not known whether the new biosimilars of ustekinumab and vedolizumab will change the therapeutic algorithm in both UC (103, 44.2%) and CD (101, 43.3%). For a significant proportion of physicians, the therapeutic algorithm will not change in both UC (56, 24.0%) and CD (61, 26.2%), while for the remainder, the therapeutic algorithm will change in both UC and CD due to a change in positioning (41, 17.6% and 40, 17.2%), greater access to therapies (13, 5.6% and 9, 3.9%), or early use of drugs (10, 4.3% and 11, 4.7%). For a quarter of physicians (62/233, 26.6%) additional research or evidence was not needed to support the use of biosimilars in IBD treatment. On the other hand, studies considered necessary for a significant percentage of respondents were studies evaluating long-term safety (34, 14.6%), head-to-head trials between originators and biosimilars (31, 13.3%), studies on multiple switches from one biosimilar to another (6, 2.6%), and studies on specific populations, such as pregnancy or perianal disease (3, 1.3%).

## 4. Discussion

To the best of our knowledge, this is the first survey specifically designed to investigate the benefits of using biosimilars for the treatment of patients with IBD. Some important insights have emerged from our work. First of all, almost all of the respondents had biosimilar drugs available in their center, indicating how their use is now standardized in clinical practice. The main reason for using biosimilars is their reduced cost, which is generally reduced by at least 30% compared to the originator drug, as confirmed by our survey. Our data are in line with the literature data and an international expert consensus [18]. Interestingly, a budget impact model revealed that the introduction of infliximab biosimilars was associated with a maximum savings of €59.4 million over 5 years [19,20]. Of note, approximately two-thirds of respondents received recommendations from their hospital pharmacy or local regulatory authority to use the lowest-cost biosimilars. This implies that the use of biosimilars is increasingly becoming a nonmedical decision. Several studies support the nonmedical switch to the biosimilar drug, demonstrating that there are no differences between originators and biosimilars in terms of clinical and biochemical remission or safety profile [21,22,23,24]. Although cost plays a key role in therapeutic choice, patient characteristics and the presence of comorbidities are essential. In fact, in patients with comorbidities, the therapeutic decision is independent of the cost of the drug. Conversely, in cases of similar efficacy and safety, cost represents the main driver of the choice. It is also necessary to underline the factors that lead to the choice of one biosimilar over others. The main discriminating factors are their availability, the cost, the characteristics of the device (autoinjector or syringe), and the hospital or insurance indications. Specifically, autoinjectors are generally preferred to syringes. Furthermore, the ease of use and grip of the device are the main characteristics that influence the choice [25]. For the healthcare system, the benefits of using biosimilars are clear and evident. However, the benefits for patients and physicians have been seldom investigated. In almost all cases, the money saved from the use of biosimilars is not reinvested to improve patient care or to improve research. This aspect certainly deserves to be explored further. In fact, the possibility of reinvesting part of the money saved through the use of biosimilars could represent an incentive for clinicians to prescribe them. On the other hand, the concept of biosimilars is not routinely explained to patients [26]. This is probably due to the fear of the nocebo effect, defined as the negative effect induced by patients’ expectations [27]. More patient information regarding biosimilars is therefore necessary and could allow an improvement in patients’ acceptance of these drugs [28]. In the near future, the patents of ustekinumab and vedolizumab will expire, and new biosimilar drugs will become available. Interestingly, for many physicians, their availability will not change the therapeutic algorithm and will not allow early access to therapies. This skepticism is probably due to greater confidence in TNF-alpha inhibitors. They are approved as a first-line therapy in both CD and UC, are extremely rapid, which justifies their use even in acute severe UC, and are recommended for the treatment of extraintestinal manifestations and challenging cases, such as perianal disease, postoperative recurrences, strictures, and specific subpopulations (e.g., pediatric patients, the elderly, and pregnant women) [6,7,8,9,10,29,30,31,32]. However, head-to-head trials are now available demonstrating how ustekinumab and adalimumab have equivalent efficacy and safety in the treatment of moderate to severe CD [33]. Similarly, in UC, vedolizumab appears to have a better performance than adalimumab [34]. Furthermore, there is strong evidence supporting the efficacy and safety of both ustekinumab and vedolizumab in multiple settings, including pregnancy, postoperative recurrence, recurrent antibiotic-refractory pouchitis, and fragile patients [10,29,35,36]. With the availability of the biosimilars of ustekinumab and vedolizumab, clinicians will finally have a broad armamentarium at their disposal. Therapeutic decisions will be effectively personalized and tailored to the specific patient and no longer limited by economic evaluation. Despite the availability of several biosimilars, not all biosimilars have been investigated in the field of IBD, and their approval has often been guided by an extrapolation mechanism from other immune-mediated disorders in order to reduce costs, avoid duplicate clinical trials, and expedite product delivery. Additional trials comparing biosimilars and reference products in IBD could ensure greater acceptability of biosimilars by clinicians and greater support from regulatory authorities. This survey has several strengths, including the large number of physicians and countries involved, which enhances the reliability and reproducibility of our results. In addition, it provides novel details about the benefits of biosimilars in IBD, including drug positioning and early access to therapies, which have never been explored before. There are also limitations to mention. First of all, the physicians who responded to the questionnaire were selected through their registration on a dedicated platform. This implies a selection bias of subjects specifically involved and interested in the field of IBD. To date, several biosimilars of adalimumab and infliximab have been approved for the management of IBD. Although their availability in each center was investigated, it was not specified which brand was practically used. Furthermore, the questionnaire was not completed in its entirety by all participants, but to overcome this problem, the total number of respondents for each question was always reported. 

## 5. Conclusions

The use of biosimilars in IBD is dictated by their reduced cost compared to originator drugs. Biosimilars of non-anti-TNF drugs will be available in the near future. However, it is not yet known whether the availability of these new biosimilars will have an impact on clinicians’ therapeutic choices and lead to a change in the therapeutic algorithm in CD and UC.

## Figures and Tables

**Table 1:** Characteristics of the survey respondents.

	*n* (%)
Specializations	
Gastroenterologists	206 (88.4)
Internists	7 (3.0)
Pediatric gastroenterologists	6 (2.6)
Surgeons	6 (2.6)
Other healthcare professionals	8 (3.4)
Experience in the field of IBD	
>10 years	179 (76.8)
5–9 years	32 (13.7)
2–4 years	19 (8.2)
≤1 year	3 (1.3)
Number of IBD patients seen per year	
<100	49 (21.0)
<500	93 (39.9)
<1000	45 (19.3)
>1000	32 (13.7)
>2000	14 (6.0)
Number of new/recently diagnosed IBD patients per year	
<50	171 (73.4)
51–100	39 (16.7)
>100	23 (9.9)

*n*: number; IBD: inflammatory bowel disease.

## Data Availability

The data that support the findings of this study are available from the corresponding author upon reasonable request.

## Supplementary Materials

The following supporting information can be downloaded at https://www.mdpi.com/article/10.3390/jcm13113069/s1, supplementary data and CROSS checklist.
