# Supplementary material for: Benefits of Biosimilars in the Management of Patients with Inflammatory Bowel Disease: An International Survey
Source: J Clin Med. 2024 May 24;13(11):3069. doi: 10.3390/jcm13113069 (PMC11172954; doi:10.3390/jcm13113069)
Supplement: Supplementary file 1 [file jcm-13-03069-s001.zip › jcm-2997482-supplementary.pdf]

Supplement data

Number of participants by country

Countries	n (%)
Italy	39 (16.7%)
Brazil	13 (5.6%)
Belgium	8 (3.4%)
Egypt	7 (3.0%)
France	7 (3.0%)
Spain	7 (3.0%)
United Kingdom	6 (2.6%)
United States	6 (2.6%)
Canada	5 (2.1%)
Germany	5 (2.1%)
Romania	5 (2.1%)
Saudi Arabia	5 (2.1%)
United Arab Emirates	5 (2.1%)
Colombia	4 (1.7%)
Croatia	4 (1.7%)
Denmark	4 (1.7%)
Israel	4 (1.7%)
Lebanon	4 (1.7%)
Poland	4 (1.7%)
Algeria	3 (1.3%)
Argentina	3 (1.3%)
Australia	3 (1.3%)
Bosnia	3 (1.3%)
Finland	3 (1.3%)
Hungary	3 (1.3%)
India	3 (1.3%)
Ireland	3 (1.3%)
Mexico	3 (1.3%)
Singapore	3 (1.3%)
South Africa	3 (1.3%)
Sweden	3 (1.3%)
Switzerland	3 (1.3%)
Turkey	3 (1.3%)

Bulgaria	2 (0.8%)
Czech Republic	2 (0.8%)
Cyprus	2 (0.8%)
Estonia	2 (0.8%)
Jordan	2 (0.8%)
Kenya	2 (0.8%)
Morocco	2 (0.8%)
Norway	2 (0.8%)
Philippines	2 (0.8%)
Portugal	2 (0.8%)
Qatar	2 (0.8%)
Ukraine	2 (0.8%)
Chile	1 (0.4%)
China	1 (0.4%)
Ecuador	1 (0.4%)
Indonesia	1 (0.4%)
Kuwait	1 (0.4%)
Latvia	1 (0.4%)
Lithuania	1 (0.4%)
New Zealand	1 (0.4%)
Nicaragua	1 (0.4%)
Russia	1 (0.4%)
Serbia	1 (0.4%)
Slovakia	1 (0.4%)
Slovenia	1 (0.4%)
Syria	1 (0.4%)
Taiwan	1 (0.4%)
Tunisia	1 (0.4%)
Venezuela	1 (0.4%)
Vietnam	1 (0.4%)

SURVEY ON BENEFITS OF BIOSIMILARS

1) Number of years of practice since your MD:

2) What country do you work in?

3) What is your specialization?
   a) Gastroenterologist
   b) Internal doctor
   c) Surgeon
   d) General practitioner
   e) Other (Please specify)

4) How many years of experience do you have in the field of IBD?
   a) Less than 1 year
   b) Less than 5 years
   c) Less than 10 years
   d) More than 10 years

5) How many IBD patients do you visit per year?
   a) Less than 100
   b) Less than 500
   c) Less than 1000
   d) More than 1000
   e) More than 2000

6) How many new/recently diagnosed IBD patients do you visit per year?

7) In your practice, is the cost of biologics a limitation for patient access to therapy?
   a. Yes
   b. No

8) Do you have biosimilars available in your practice?
   a) Yes
   b) No

9) If you do not have biosimilars available in your practice, what is the main reason not having biosimilars?

10) If you have biosimilars available in your practice, please specify which biosimilars you have
   a. Infliximab
   b. Adalimumab
   c. Both infliximab and adalimumab

11) Do you have more than one adalimumab biosimilar available?
   a. Yes

b. No

12) Do you have more than one infliximab biosimilar available?
    a. Yes
    b. No

13) If you have more than one infliximab and/or adalimumab biosimilar available, what kind of approach do you use to make a choice between available biosimilars for a concrete patient?

14) In your practice, do adalimumab biosimilars cost less than the originator drug?
    a. Yes
    b. No

15) If adalimumab biosimilars cost less than the originator drug, how much less do they cost?
    a. <10%
    b. 10-20%
    c. 20-30%
    d. 30-50%
    e. 50-70%
    f. >70%

16) In your practice, do infliximab biosimilars cost less than the originator drug?
    a. Yes
    b. No

17) If infliximab biosimilars cost less than the originator drug, how much less do they cost?
    g. <10%
    h. 10-20%
    i. 20-30%
    j. 30-50%
    k. 50-70%
    l. >70%

18) Does your department receive a reimbursement if you use biosimilars instead of originator drugs (multiple answers allowed)?
    a. Yes, I can you use this money to improve patient care
    b. Yes can you use this money to improve research
    c. No
    d. Other (please speficy)

19) Does your hospital pharmacy provide recommendations regarding the prescription of biosimilars or originator drugs?
    a. Yes
    b. No

20) If your hospital pharmacy provides recommendations regarding the prescription of biosimilars or originator drugs, please specify the type of recommendation.

21) Does your local regulatory authority provide recommendations regarding the prescription of biosimilars or originator drugs?
    a. Yes
    b. No

22) If your local regulatory authority provides recommendations regarding the prescription of biosimilars or originator drugs, please specify the type of recommendation.

23) If your patients know what a biosimilar is, what is the main source for their awareness?

24) What percentage of your patients know what a biosimilar is?
    a. <10%
    b. 11-25%
    c. 26-50%
    d. 51-75%
    e. >75%

25) In your opinion, what are the potential benefits of biosimilars for IBD patients?
    a. Reduced healthcare costs
    b. Improved access to treatment
    c. Increased treatment options
    d. Increased competition leading to improved innovation

26) Do you explain the benefits of biosimilars to your patients?
    a. Systematically
    b. Often
    c. Sometimes
    d. Never

27) If you explain the benefits of biosimilars to your patients, how much do your patients understand and support the benefits of biosimilars from 0 to 10 (0=very little; 10=fully)?

28) Has the availability of biosimilars allowed you to treat more patients in your practice?
    a. Yes
    b. No

29) Has the availability of biosimilars allowed you to treat patients earlier?
    a. Yes
    b. No

30) In your practice, when is the switch from the originator drug to the biosimilar performed?
    a. At any time, but it is a medical decision
    b. When the patient is in remission
    c. It is a non-medical decision
    d. Other (please specify)

31) How the availability of biosimilars can influence to your treatment decisions in Crohn's disease?

32) How the availability of biosimilars can influence to your treatment decisions in ulcerative colitis?

33) Which disease characteristics influence your decision to use a biosimilar over the originator drug (multiple answers allowed)?
    a. Disease duration
    b. Disease complications
    c. Disease location
    d. Disease severity
    e. Previous treatment
    f. Other (please specify)
    g. They do not influence my decision

34) In an IBD patient naïve to advanced therapies and without comorbidities, how much does the therapeutic decision between an adalimumab/infliximab biosimilar and an originator drug of another mechanism of action (e.g. ustekinumab or vedolizumab) depend on the cost from 0 to 10 (0=minimum; 10=maximum)?

35) In an IBD patient naïve to advanced therapies with comorbidities, how much does the therapeutic decision between an adalimumab/infliximab biosimilar and an originator drug of another mechanism of action (e.g. ustekinumab or vedolizumab) depend on the cost from 0 to 10 (0=minimum; 10=maximum)?

36) In a patient with IBD already treated with advanced therapies and without comorbidities, how much does the therapeutic decision between an adalimumab/infliximab biosimilar and an originator drug of another mechanism of action (e.g. ustekinumab or vedolizumab) depend on cost from 0 to 10 (0=minimum; 10=maximum)?

37) In a patient with IBD already treated with advanced therapies and with comorbidities, how much does the therapeutic decision between an adalimumab/infliximab biosimilar and an originator drug of another mechanism of action (e.g. ustekinumab or vedolizumab) depend on cost from 0 to 10 (0=minimum; 10=maximum)?

38) How the availability of ustekinumab and vedolizumab biosimilar in the near future will increase access to therapies?

39) How the availability of ustekinumab and vedolizumab biosimilar in the near future will influence the therapeutic algorithm in UC?

40) How the availability of ustekinumab and vedolizumab biosimilar in the near future will influence the therapeutic algorithm in CD?

41) Do you think the availability of biosimilars will allow more patients to be treated in an earlier stage of their disease?

42) What additional research or evidence is needed to support the use of biosimilars in IBD treatment?

Supplementary Table . CROSS checklist

Section/topic	Item	Item description	Reported on page #
**Title and abstract**			
Title and abstract	1a	State the word "survey" along with a commonly used term in title or abstract to introduce the study's design.	1
	1b	Provide an informative summary in the abstract, covering background, objectives, methods, findings/results, interpretation/discussion, and conclusions.	1
**Introduction**			
Background	2	Provide a background about the rationale of study, what has been previously done, and why this survey is needed.	1-2
Purpose/aim	3	Identify specific purposes, aims, goals, or objectives of the study.	2
**Methods**			
Study design	4	Specify the study design in the methods section with a commonly used term (e.g., cross-sectional or longitudinal).	2
Data collection methods	5a	Describe the questionnaire (e.g., number of sections, number of questions, number and names of instruments used).	2
	5b	Describe all questionnaire instruments that were used in the survey to measure particular concepts. Report target population, reported validity and reliability information, scoring/classification procedure, and reference links (if any).	2
	5c	Provide information on pretesting of the questionnaire, if performed (in the article or in an online supplement). Report the method of pretesting, number of times questionnaire was pre-tested, number and demographics of participants used for pretesting, and the level of similarity of demographics between pre-testing participants and sample population.	2
	5d	Questionnaire if possible, should be fully provided (in the article, or as appendices or as an online supplement).	Yes, in Data Suppl.
Sample characteristics	6a	Describe the study population (i.e., background, locations, eligibility criteria for participant inclusion in survey, exclusion criteria).	2
	6b	Describe the sampling techniques used (e.g., single stage or multistage sampling, simple random sampling, stratified sampling, cluster sampling, convenience sampling). Specify the locations of sample participants whenever clustered sampling was applied.	
	6c	Provide information on sample size, along with details of sample size calculation.	NA
	6d	Describe how representative the sample is of the study population (or target population if possible), particularly for population-based surveys.	NA
Survey	7a	Provide information on modes of questionnaire administration, including the type and number of contacts, the location where the survey was conducted (e.g., outpatient room	2

administration		or by use of online tools, such as SurveyMonkey).	
	7b	Provide information of survey’s time frame, such as periods of recruitment, exposure, and follow-up days.	2
	7c	Provide information on the entry process:<br>–>For non-web-based surveys, provide approaches to minimize human error in data entry.<br>–>For web-based surveys, provide approaches to prevent “multiple participation” of participants.	2
Study preparation	8	Describe any preparation process before conducting the survey (e.g., interviewers’ training process, advertising the survey).	NA
Ethical considerations	9a	Provide information on ethical approval for the survey if obtained, including informed consent, institutional review board [IRB] approval, Helsinki declaration, and good clinical practice [GCP] declaration (as appropriate).	NA
	9b	Provide information about survey anonymity and confidentiality and describe what mechanisms were used to protect unauthorized access.	2
Statistical analysis	10a	Describe statistical methods and analytical approach. Report the statistical software that was used for data analysis.	NA
	10b	Report any modification of variables used in the analysis, along with reference (if available).	NA
	10c	Report details about how missing data was handled. Include rate of missing items, missing data mechanism (i.e., missing completely at random [MCAR], missing at random [MAR] or missing not at random [MNAR]) and methods used to deal with missing data (e.g., multiple imputation).	2
	10d	State how non-response error was addressed.	NA
	10e	For longitudinal surveys, state how loss to follow-up was addressed.	NA
	10f	Indicate whether any methods such as weighting of items or propensity scores have been used to adjust for non-representativeness of the sample.	NA
	10g	Describe any sensitivity analysis conducted.	NA
**Results**			
Respondent characteristics	11a	Report numbers of individuals at each stage of the study. Consider using a flow diagram, if possible.	2
	11b	Provide reasons for non-participation at each stage, if possible.	NA
	11c	Report response rate, present the definition of response rate or the formula used to calculate response rate.	2-5
	11d	Provide information to define how unique visitors are determined. Report number of unique visitors along with relevant proportions (e.g., view proportion, participation proportion, completion proportion).	2-5

Descriptive results	12	Provide characteristics of study participants, as well as information on potential confounders and assessed outcomes.	2-5
	13a	Give unadjusted estimates and, if applicable, confounder-adjusted estimates along with 95% confidence intervals and p-values.	NA
Main findings	13b	For multivariable analysis, provide information on the model building process, model fit statistics, and model assumptions (as appropriate).	NA
	13c	Provide details about any sensitivity analysis performed. If there are considerable amount of missing data, report sensitivity analyses comparing the results of complete cases with that of the imputed dataset (if possible).	NA
**Discussion**			
Limitations	14	Discuss the limitations of the study, considering sources of potential biases and imprecisions, such as non-representativeness of sample, study design, important uncontrolled confounders.	6
Interpretations	15	Give a cautious overall interpretation of results, based on potential biases and imprecisions and suggest areas for future research.	5,6
Generalizability	16	Discuss the external validity of the results.	5,6
**Other sections**			
Role of funding source	17	State whether any funding organization has had any roles in the survey's design, implementation, and analysis.	7
Conflict of interest	18	Declare any potential conflict of interest.	7
Acknowledgements	19	Provide names of organizations/persons that are acknowledged along with their contribution to the research.	7
